# Giant tunable spin Hall angle in sputtered Bi_2_Se_3_ controlled by an electric field

**DOI:** 10.1038/s41467-022-29281-w

**Published:** 2022-03-28

**Authors:** Qi Lu, Ping Li, Zhixin Guo, Guohua Dong, Bin Peng, Xi Zha, Tai Min, Ziyao Zhou, Ming Liu

**Affiliations:** 1 Electronic Materials Research Laboratory, Key Laboratory of the Ministry of Education & International Center for Dielectric Research, School of Electronic and Information Engineering, State Key Laboratory for Manufacturing System Engineering, Xi’an Jiaotong University, 710049 Xi’an, China; 2 Center for Spintronics and Quantum System, State Key Laboratory for Mechanical Behavior of Materials, School of Materials Science and Engineering, Xi’an Jiaotong University, 710049 Xi’an, China

**Keywords:** Electronic devices, Spintronics

## Abstract

Finding an effective way to greatly tune spin Hall angle in a low power manner is of fundamental importance for tunable and energy-efficient spintronic devices. Recently, topological insulator of Bi_2_Se_3_, having a large intrinsic spin Hall angle, show great capability to generate strong current-induced spin-orbit torques. Here we demonstrate that the spin Hall angle in Bi_2_Se_3_ can be effectively tuned asymmetrically and even enhanced about 600% reversibly by applying a bipolar electric field across the piezoelectric substrate. We reveal that the enhancement of spin Hall angle originates from both the charge doping and piezoelectric strain effet on the spin Berry curvature near Fermi level in Bi_2_Se_3_. Our findings provide a platform for achieving low power consumption and tunable spintronic devices.

## Abstract (web summary)

Controlling the spin Hall angle is significant to tunable and energy-efficient spintronic devices. Here, the authors demonstrate that the spin Hall angle in Bi_2_Se_3_ can be tuned and even enhanced about 600% reversibly by the electric field.

## Introduction

Driving magnetization switching by current-induced spin-orbit torque (SOT) is one of the most promising means to achieve magnetic random access memories (MRAM) due to the merits in ultrafast, nonvolatile, and energy-efficient operation^1^. In a typical SOT-driven device, the in-plane charge current is converted into spin current in the heavy metal layer via the spin Hall effect^2^. The spin current further injects into the adjacent ferromagnetic layer, exerting torque on the magnetic moment and driving magnetization switching. The power consumption of SOT-MRAM devices can be further reduced by improving the charge-spin conversion efficiency in the nonmagnetic spin Hall layer^3–5^ or enhancing the spin current transmission at the nonmagnet/ferromagnet interface^5–7^. Here, the spin Hall angle defined as the ratio of spin current density *J*_s_ to charge current density *J*_c_ is crucial in SOT devices. Pursuing materials with a large spin Hall angle is a prerequisite for achieving low power consumption SOT devices. In this context, topological insulators^8^, 2D materials^9^, antiferromagnets^10^, and Weyl semimetals^11–13^ with novel quantum states have been actively investigated. Topological insulators such as Bi_2_Se_3_ and Bi_2_Te_3_ show an exceptionally large spin-charge conversion efficiency than conventional heavy metals due to the helical spin-momentum locking in the surface states^14–16^. Recently, magnetron sputtered Bi_2_Se_3_ films exhibiting both large spin Hall angle and highly compatible with industrial production, are regarded as optimal materials for the SOT switching^17,18^. Despite these exciting prospects, topological insulators are problematic because of their relative high resistivities. Exploring interesting approaches to further improve energy efficiency is a challenge and opportunity.

The electric field-induced mechanical strain has been demonstrated to effectively mediate the magnetic anisotropy, exchange coupling, spin structure, and SOT magnitude in the ferromagnetic layer^19–22^. In addition, the surface charge doping related to the ferroelectric polarization will also contribute to the tuning effect. Due to the dielectric nature of ferroelectric materials, the electric field can be applied without the leak current. Hence the Joule heating is suppressed in electric field controlling, which is suitable for low energy consuming information technology. However, in the spin Hall materials, the electric field control of the charge-spin conversion ratio has been rarely studied. Considering that the intrinsic spin Hall conductivities are strongly dependent on the position of Fermi level, which can be tuned by the charge doping and strain. The strain effect usually shows a symmetric tunning effect with respect to the positive and negative electric fields due to the same lattice deformation, while the charge doping effect always shows an asymmetric tunning effect^23^. Thus building the multifunctional hybrid devices by introducing electric field controlling is particularly attractive to obtaining tunable spin sources and further reducing the power consumption of SOT-MRAM devices.

In this work, the electric field dramatic tuning of spin Hall angle in Bi_2_Se_3_ films sputtered on the piezoelectric substrates was demonstrated. The values of spin Hall angle under different electric fields are measured using an in situ planar Hall system. With applying an electric field, the charge-spin conversion efficiency was significantly enhanced in a reversible and reproducible manner. The maximum spin Hall angle was determined to be 13.45 at *E* = −12 kV/cm which is six times larger than the initial state. It is interesting to find the spin Hall angle changed asymmetrically while a bipolar electric field was applied across the piezoelectric substrate. By conducting DFT calculation and related control experiments, such great tunability as well as the asymmetrical tuning effect are considered to be originating from the combination effect of the mechanical strain and the interfacial charge doping between Bi_2_Se_3_ and PMN-PT. It turns out the mechanical strain enhances the spin Hall angle symmetrically, whereas the charge doping changes the spin Hall angle in an asymmetrical way.

## Results

**Fig. 1:**
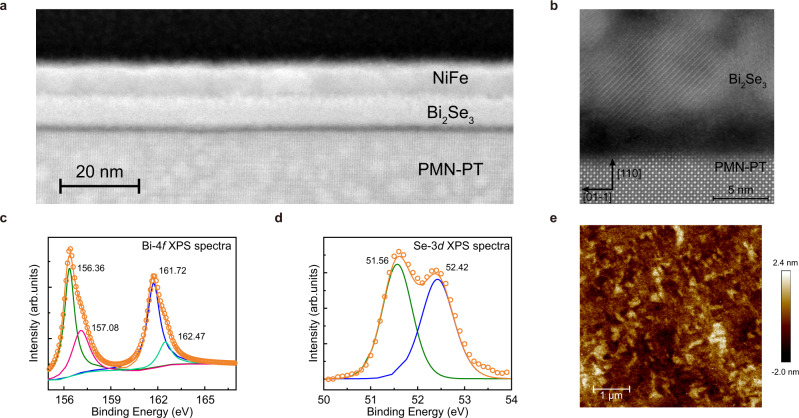
Structure, element and surface morphology characterization of the film. **a** Low magnification and (**b**) high-resolution cross-sectional HAADF-STEM image of PMN-PT/Bi_2_Se_3_/NiFe. The XPS spectra for the Bi_2_Se_3_ film where (**c**) Bi-4*f* core level and (**d**) Se-3*d* core level. **e** AFM morphology of PMN-PT/Bi_2_Se_3_/NiFe sample.

The low magnification high-angle annular dark-field scanning transmission electron microscopy (HAADF-STEM) image gives an overview of the PMN-PT/Bi_2_Se_3_(8 nm)/NiFe(8 nm)/TaO_*x*_(1 nm) structure, as shown in Fig. 1a. The thickness of the layers is similar to the nominal ones. The interface of each layer is smooth and continuous, which indicating the uniform growth of all layers in the stack (see Supplementary Fig. 6). Figure 1b shows the high-resolution HAADF-STEM image of the film. The sharp lattice fringes clearly seen in the image reveal the existence of good crystallinity in the Bi_2_Se_3_. The Bi_2_Se_3_ film exhibits a mixing of polycrystalline and amorphous state as a whole (see Supplementary Fig. 7). This result is consistent with the previous report on the sputtered Bi_2_Se_3_ films^17,24^. We conducted the X-ray photoelectron spectroscopy (XPS) measurement to determine the chemical composition of PMN-PT/Bi_2_Se_3_ (20 nm) structure. Figure 1c, d shows the Bi 4*f* core-level spectra and Se 3*d* core-level spectra, respectively. For Bi 4*f* core-level spectra, the peaks of Bi 4*f*_7/2_ and 4*f*_5/2_ energy levels are observed at binding energies about 156.36 eV and 161.72 eV. The Bi 4*f* levels show a spin-orbit splitting of 5.36 eV, which is consistent with the previous report^25^. Here two other components with the binding energy of 157.08 eV and 162.47 eV nearby the original Bi 4*f* peaks are observed. Those peaks corresponding to the Bi-O bound are due to the little oxidation when measuring the XPS spectra^26^. Two peaks correspond to the Se 3*d*_5/2_, and 3*d*_3/2_ with binding energies of 51.56 eV and 52.42 eV appear at the Se 3*d* core-level spectra. The splitting of Se 3*d* level is determined to be 0.86 eV, comparable to the previous experimental studies^18,27,28^. By considering the integrated peak intensity with the sensitivity factor, the surface atomic composition ratio of Se and Bi is estimated to be 1.43 (see Supplementary Fig. 8 and Supplementary Note 7). The composition is close to the target, showing the films are normally stoichiometric. To examine the surface roughness of the samples, we conducted the atomic force microscopy (AFM) measurement on the PMN-PT/Bi_2_Se_3_(8 nm)/NiFe(8 nm)/TaO_*x*_(1 nm) sample, as shown in Fig. 1e. The surface roughness Ra is determined to be 0.489 nm, indicating a smooth surface and high film quality which is sufficient for the further SOT study (see Supplementary Fig. 10).

**Fig. 2:**
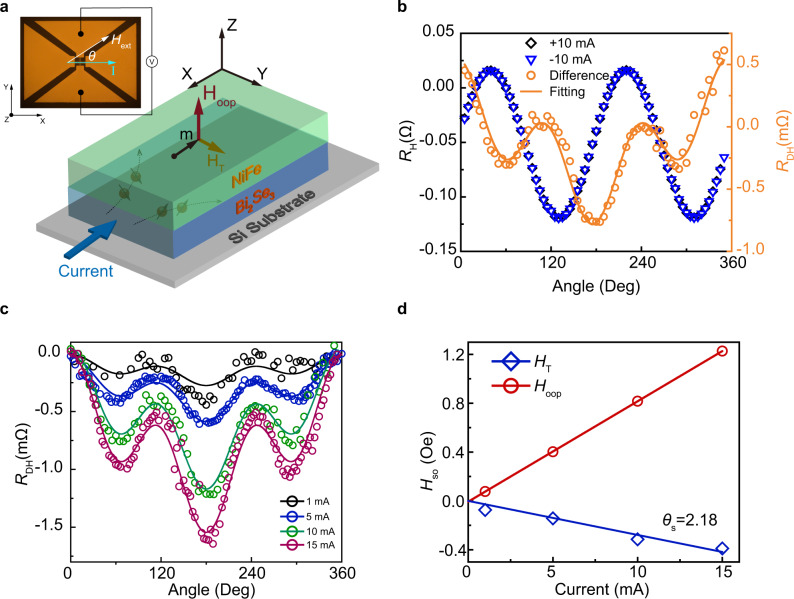
Determine the SOT-induced effective field and spin Hall angle by planar Hall effect. **a** Schematic for the coordinate system and the SOT-induced effective fields considered in the NiFe/Bi_2_Se_3_ heterostructure. The inset is a top view of the Hall device by optical microscopy and the layout for the planar Hall measurement. **b** Planar Hall resistance *R*_H_ of NiFe/Bi_2_Se_3_ at ±10 mA applied current as a function of the external magnetic field angle *θ*. The right axis represents the difference of the Hall resistances *R*_DH_ at positive and negative currents. **c** Angular-dependent *R*_DH_ measured at different input currents. Each solid curve represents the fitting result. **d** SOT-induced effective fields *H*_OOP_ and *H*_T_ as a function of applied current. The lines are the linear fitting results.

Here we determine the current-induced SOT effective fields by angular field characterization of the planar Hall resistance at positive and negative testing current. The damping-like torque induces the perpendicular effective field *H*_OOP_, and the field-like torque induces the in-plane transverse effective field *H*_T_. as shown in Fig. 2a. According to the Slonczewski equation^29^, the perpendicular effective field *H*_OOP_ can be expressed as $-[\hslash /(2e{M}_{{{{{{\rm{S}}}}}}}t)]{J}_{{{{{{\rm{S}}}}}}}({{{{{\boldsymbol{\sigma }}}}}}\times {{{{{\boldsymbol{m}}}}}})$, where ***σ*** is the spin polarization unit vector, ***m*** is the magnetization vector, *J*_s_ represent the magnitude of spin current density, and *t* is the thickness of the ferromagnetic layer. The Hall resistance is directly modified by the small magnetization deviation induced by the SOT effective fields. The direction of the *H*_oop_ and *H*_T_ relies on the sign of current. Hence the component of SOT effective fields in angular-dependent planar Hall resistance is an odd function of current, whereas other components are even function with respect to the testing current. We take the differential resistance ${R}_{{{{{{\rm{DH}}}}}}}={R}_{{{{{{\rm{H}}}}}}}(+I)-{R}_{{{{{{\rm{H}}}}}}}(-I)$ to extract the contribution from the SOT effective fields and cancel out other effects such as Joule heating^30^. The inset illustrates the structure of the Hall device and the measurement configuration. The Hall resistance *R*_H_(*I*,*θ*) is measured using the bipolar input current and rotating the constant 400 Oe in-plane external magnetic field *H*_ext_. Here *I* is the input current along the *x*-direction, and *θ* represents the angle between the current and the magnetic field.

Figure 2b shows the typical planar Hall resistance *R*_H_ as a function of *θ* measured at ±10 mA and the corresponding *R*_DH_ curve for Bi_2_Se_3_(8 nm)/NiFe(8 nm)/TaO_*x*_(1 nm) structure on thermally oxidized Si substrate. Here we assume that the top 1 nm Ta capping layer is fully oxidized and has almost no impact on the spin transport properties^31,32^. The value of *H*_OOP_ and *H*_T_ can be obtained by fitting the following equation

1 $${R}_{{{{{{\rm{DH}}}}}}}(I,\theta )\,=	\, 2{R}_{{{{{{\rm{H}}}}}}}\frac{({H}_{{{{{{\rm{T}}}}}}}+{H}_{{{{{{\rm{Oe}}}}}}})}{{H}_{{{{{{\rm{ext}}}}}}}}(\cos \theta +\,\cos 3\theta )\\ 	+2\frac{{{{{{\rm{d}}}}}}{R}_{{{{{{\rm{AHE}}}}}}}}{{{{{{\rm{d}}}}}}{H}_{{{{{{\rm{perp}}}}}}}}{H}_{{{{{{\rm{OOP}}}}}}}\,\cos \theta +C$$

where *C* is the resistance offset, *R*_AHE_ is the anomalous Hall resistance, and *H*_perp_ is the magnetic field along the *z* direction when measuring the anomalous Hall resistance. The Oersted field contribution *H*_Oe_ is estimated to be 6.66 × 10^−3^ Oe/mA calculated by Ampere’s law with considering the current shutting effect (see Supplementary Note 9). The *R*_H_ is the magnitude of sin(2*θ*) component in the corresponding planar Hall resistance curve. Here the value of $\frac{{{{{{\rm{d}}}}}}{R}_{{{{{{\rm{AHE}}}}}}}}{{{{{{\rm{d}}}}}}{H}_{{{{{{\rm{perp}}}}}}}}$ is determined to be 3.175 × 10^−5^ Ω/Oe by taking the slope of *R*_AHE_ versus *H*_perp_ at a small 1 mA testing current (see Supplementary Note 3).

After determining the value of *R*_H_, *H*_Oe_, and $\frac{{{{{{\rm{d}}}}}}{R}_{{{{{{\rm{AHE}}}}}}}}{{{{{{\rm{d}}}}}}{H}_{{{{{{\rm{perp}}}}}}}}$ parameters, the effective field *H*_OOP_ and *H*_T_ can be obtained by fitting the angular-dependent *R*_DH_ data into Eq. (1). Figure 2c shows the *R*_DH_ results and the fitting curves at different testing currents. The fitting curves are in good agreement with the experimental data. The amplitude of *R*_DH_ curve increases with increasing the testing current, indicating the contribution from the current-induced SOT effective fields. Figure 2d shows the linear fitting result of SOT effective field *H*_SO_, including *H*_OOP_ and *H*_T_ with respect to the testing current. The effective spin Hall angle can be calculated by

2 $${\theta }_{{{{{{\rm{S}}}}}}}=\frac{2e{M}_{{{{{{\rm{S}}}}}}}{t}_{{{{{{\rm{FM}}}}}}}}{\hslash }\frac{{H}_{{{{{{\rm{OOP}}}}}}}}{{J}_{{{{{{\rm{NM}}}}}}}}$$

where *M*_s_ is the saturation magnetization and equal to 663.45 emu/cm^3^ (see Supplementary Fig. 4), *t*_FM_ is the thickness of the ferromagnetic layer, and *J*_NM_ is the current density in the nonmagnetic layer. The value of $\frac{{H}_{{{{{{\rm{OOP}}}}}}}}{{J}_{{{{{{\rm{NM}}}}}}}}$ is obtained by the slope of the linear fitting result. The spin Hall angle of our 8 nm Bi_2_Se_3_ is determined to be 2.18, comparable with the previous study on sputtered Bi_2_Se_3_ and the ST-FMR analysis (see Supplementary Note 1). This ensures that the measurements and analysis of spin Hall angle are performed precisely and reliable.

**Fig. 3:**
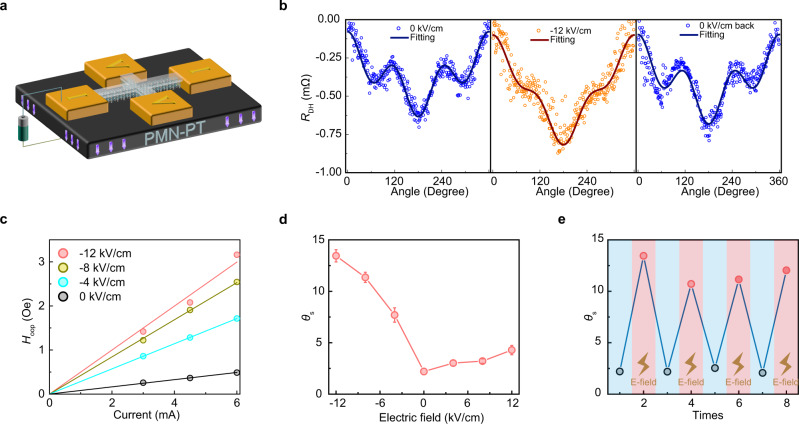
In situ voltage controls spin Hall angle. **a** Schematics of the experimental setup for the voltage control spin Hall angle. **b** Typical angular-dependent *R*_DH_ measured at the electric field from 0 kV/cm to −12 kV/cm, and then back to 0 kV/cm with the fitting results. **c** The obtained effective field *H*_OOP_ under different electric fields with the linear fitting results versus the applied current. **d** The spin Hall angle of Bi_2_Se_3_ as a function of the external electric field. Error bars represent standard deviation. **e** The reversibility test of the electric field control process.

We perform the in situ planar Hall measurements at different electric fields to characterize the electric field control spin Hall angle of Bi_2_Se_3_. The Hall device with the same multilayer structure is fabricated on a PMN-PT substrate that applies the electric field and generates mechanical strain, as demonstrated in Fig. 3a. The electric field is applied perpendicular to the PMN-PT substrate up to ±12 kV/cm. The representative *R*_DH_ results measuring at ±6 mA testing current under 0 kV/cm, −12 kV/cm, and 0 kV/cm back electric field are shown in Fig. 3b. The constant baseline of the results is substracted to directly compare the amplitude and line shape of the *R*_DH_ curves under different electric fields. The *R*_DH_ curves under −12 kV/cm exhibit a larger amplitude than that of 0 kV/cm, indicating the increment of the SOT-induced effective fields. Specifically, the peaks around 115° and 245° nearly disappear under the electric field at −12 kV/cm. Such dramatic change in the line shape is due to an increase of the cos(*θ*) component to the total line shape, which corresponds to a remarkable enhancement of perpendicular effective field *H*_OOP_. Note that there are no obvious changes in the *R*_DH_ curves between 0 kV/cm and 0 kV/cm back, implying a reversible switching behavior.

The *H*_OOP_ versus the testing current from 0 kV/cm to −12 kV/cm is presented in Fig. 3c. The determined effective fields are proportional to the testing current, and the linear fitting agrees with the data well. The values of $\frac{{H}_{{{{{{\rm{OOP}}}}}}}}{{J}_{{{{{{\rm{NM}}}}}}}}$ derived from the linear fitting are 1.227 × 10^−9^ Oe/(A m^−2^) and 7.976 × 10^−9^ Oe/(A m^−2^) under 0 kV/cm to −12 kV/cm, respectively. Figure 3d demonstrates the spin Hall angles under different electric fields applied across the PMN-PT substrate. Interestingly, it is found that the electric field-dependent spin Hall angle is significantly asymmetric with respect to the zero field. The spin Hall angle of Bi_2_Se_3_ has been enhanced by using both positive and negative electric fields. Especially, the spin Hall angle of Bi_2_Se_3_ exhibits a significant increment up to 13.45 under −12 kV/cm, which is about 6 times larger than the initial state.

**Fig. 4:**
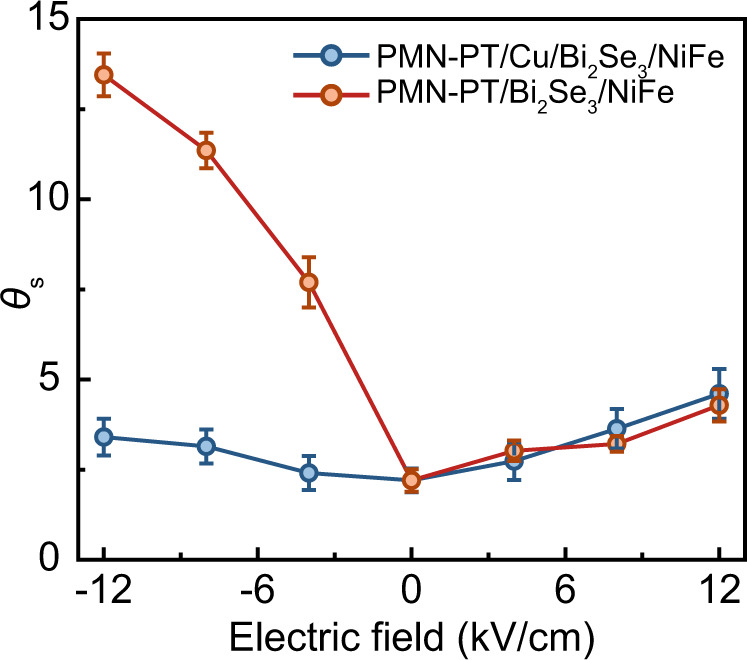
Comparison of electric field control spin Hall angle under the conditions with or without Cu dissipation layer between PMN-PT substrate and Bi_2_Se_3_ layer. Error bars represent standard deviation.

Generally speaking, two effects from the PMN-PT substrate are expected to be responsible for the change of the spin Hall angle in Bi_2_Se_3_, i.e., strain effect^19,33^ and charge doping effect^23,34^. In order to clarify the mechanism of the tunning result, it is necessary to directly distinguish the strain effect and charge doping effect. Inserting a charge dissipation layer such as 5 nm Cu between PMN-PT substrate and Bi_2_Se_3_ layer is an effective way to separate those two effect^34,35^. The charge doping effect can be excluded in this condition since the charge will be dissipated in the Cu layer. Here we further perform the same study with structure PMN-PT/Cu(5 nm)/Bi_2_Se_3_(8 nm)/NiFe(8 nm)/TaO_*x*_(1 nm). Figure 4 gives a comparison of electric field control spin Hall angle between PMN-PT/Bi_2_Se_3_/NiFe structure and PMN-PT/Cu/Bi_2_Se_3_/NiFe structure. After inserting the Cu dissipation layer between Bi_2_Se_3_ and PMN-PT, the electric field control spin Hall angle in Bi_2_Se_3_ shows a symmetry behavior at positive and negative electric fields. That is due to the almost equivalent lattice deformation induced by the piezoelectric strain^36^. After separating the strain effect, the higher enhancement of spin Hall angle in PMN-PT/Bi_2_Se_3_/NiFe structure at the negative electric field can be attributed to the contribution of the charge doping effect.

To theoretically understand the tunning mechanism, we further investigate the external electric field effect on the spin Hall angle of Bi_2_Se_3_ by first-principle calculation. The spin Hall conductivity (SHC) of Bi_2_Se_3_ which is proportional to the spin Hall angle, as a function of in-plane strain and charge doping have been studied respectively.

**Fig. 5:**
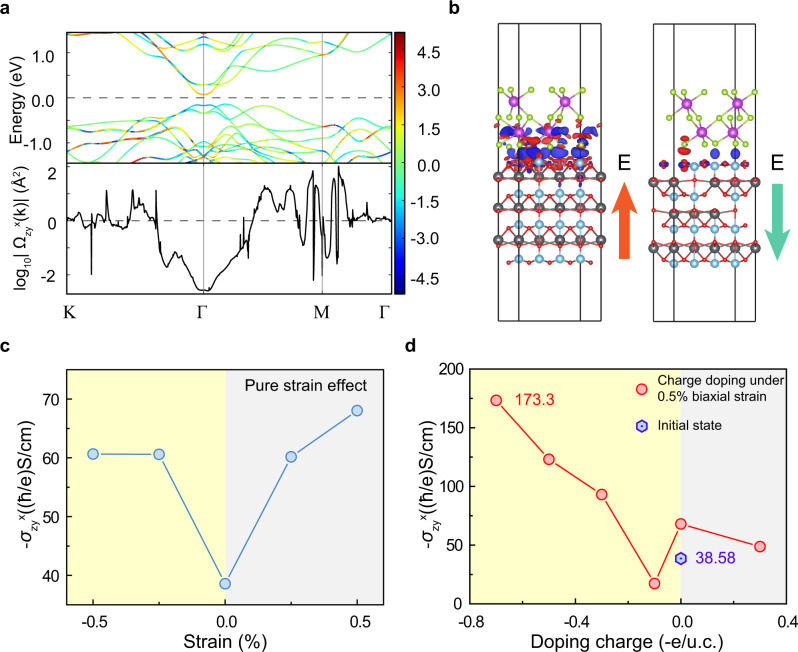
Spin Hall conductivity calculation for Bi_2_Se_3_ structure. **a** Band structure projected by spin Berry curvature on a log scale (up panel) and the k-resolved spin Berry curvatures (down panel) for the 0.5% biaxial strain bulk Bi_2_Se_3_. **b** Charge density difference of heterostructure of Bi_2_Se_3_ on TiO-terminal PbTiO_3_(001) surface. The picture on the left shows the application of the negative external electric field (along the *z* direction) on the heterostructure. Simultaneously, the built-in electric field along the −*z* direction is generated in PbTiO_3_. The polarization of PbTiO_3_ is P↑. The picture on the right is the opposite. The polarization of PbTiO_3_ is P↓. The red and blue areas indicate loss and gain electrons, respectively. The Pb, Ti, O, Bi, and Se atoms are depicted by gray, light blue, red, magenta, and green balls, respectively. **c** SHC as a function of the biaxial strain for bulk Bi_2_Se_3_. **d** SHC as a function of the charge doping for the bulk Bi_2_Se_3_ under 0.5% biaxial strain. The initial state of Bi_2_Se_3_ without strain and charge doping is marked with the blue hexagon symbol.

We adopt the maximally localized Wannier functions with Wannier90 in combination with the VASP package to calculate the SHC^37–40^. Firstly, we calculate the SHC of bulk Bi_2_Se_3_ with R-3m space group and find its SHC is 38.58 (*ħ*/*e*)S/cm, which is mainly attributed to the electrons around the Fermi level nearby the Γ point in the Brillouin zone (Fig. 5a). It is known that the electric field induces biaxial strain in Bi_2_Se_3_ via PMN-PT substrate^41^. Thus, we systematically investigate the relationship between SHC of Bi_2_Se_3_ and the biaxial strain. As shown in Fig. 5c, both the tensile and compressive strains can induce the increment of SHC. Moreover, the tensile and compressive strains tune the SHC almost symmetric when the strain is small (<0.25%). When the strain becomes larger, i.e., to the maximum value of our experimental data 0.5%, the asymmetric SHC appears. Nevertheless, the asymmetric difference is less than 20%, much smaller than the experimental results where three times asymmetric is observed (Fig. 3d). This result is consistent with the experimental observation where the charge transfer effect is excluded (Fig. 4).

To further explore the charge transfer effect induced by the PMN-PT substrate, we additionally calculate the electronic structure of a heterostructure composed of Bi_2_Se_3_ and PbTiO_3_(001) (a simplified model to PMN-PT). Considering that the optimized lattice constant of Bi_2_Se_3_ is 4.16 Å and that of PbTiO_3_ is 3.90 Å, the $1\times \sqrt{7}$ unit cell of Bi_2_Se_3_ is commensurate to the $1\times 2\sqrt{2}$ surface unit cell of PbTiO_3_(001). Figure 5b shows the calculated differential charge density distributions for TiO-terminal Bi_2_Se_3_/PbTiO_3_(001) heterostructure. It is seen that obvious electron transfer from PbTiO_3_ to Bi_2_Se_3_ occurs when PbTiO_3_ is under the negative electric field, whereas there is almost no electron transfer when the electric field is switched to positive. Note that the above results have been confirmed by the DFT calculations based on the HSE06 functional^42,43^, which is well known to give rise to reliable band gaps in semiconductors (see Supplementary Fig. 11). In addition, we have considered the vacancy defect effect in Bi_2_Se_3_ on the interface charge transfer and calculated the differential charge density of Se vacancy defect at the interface (see Supplementary Fig. 12). As one can see, the Se vacancy defect does not affect the charge transfer from PbTiO_3_ to Bi_2_Se_3_. Moreover, the effect of metallic covering layer (NiFe alloy) on Bi_2_Se_3_ interface charge transfer has been investigated. Here we simply consider the Fe/Bi_2_Se_3_ heterostructure, due to the similar effect on the charge transfer between Ni and Fe elements. The charge transfer from PbTiO_3_ to Bi_2_Se_3_ still occurs when the negative electric field is applied. Whereas, when the electric field is reversed, there is almost no charge transfer between PbTiO_3_ and Bi_2_Se_3_. This result is similar to the case without the metallic covering layer. We have further calculated the averaged charge density difference along the out-of-plane *z* direction denoted as Δ*ρ*(*z*) by integrating it over the *x*–*y* plane (see Supplementary Fig. 13). It is found that the Se atoms at the interface have a nature of obtaining electrons from Fe atoms when no external electric field is applied. It should be noted that the negative external electric field further benefits the electron transfer from the Fe layer to Bi_2_Se_3_. These results confirm that the additional electrons transferred from PMN-PT to Bi_2_Se_3_ can be preserved in Bi_2_Se_3_.

We have then calculated the SHC of bulk Bi_2_Se_3_ as a function of the charge doping concentration (-e/unit-cell) under 0.5% biaxial strain. As shown in Fig. 5d, the SHC significantly increases with the increase of doped  charge concentration from −0.1 to −0.7 -e/unit-cell, but it decreases from 0 to 0.3 -e/unit-cell. Such a significant increase of SHC with charge doping can be due to the upshift of the Fermi level, which involves the contribution of conduction electrons around the Γ point to the spin Berry curvature. Especially, the SHC becomes about three times as that without electron transfer under 0.5% strain when the charge doping is about −0.7 -e/unit-cell. These results show that the asymmetry of the spin Hall angle is mainly from the charge transfer effect. In addition, compared to that of the initial state without strain and charge doping, the coordinated effect of strain and charge doping can increase the SHC of Bi_2_Se_3_ by 4.5 times in our DFT calculations, which is close to the experimental observation (6 times).

## Discussion

In conclusion, we have demonstrated a giant (more than 600%) and reversible manipulation of spin Hall angle in sputtered Bi_2_Se_3_ films solely by electric fields across the PMN-PT piezoelectric substrate. The spin Hall angle and SOT effective field are evaluated using the angular-dependent planar Hall measurement with the in situ electric field. We find that applying an electric field enhances the spin Hall angle from 2.18 to 13.45. Moreover, we conduct the first-principles calculation study on the intrinsic spin Hall conductivity in a relevant system to reveal the modulation mechanism. We find this remarkable modulation of spin Hall angle is achieved by the co-mediating of electric-field-induced strain and surface charge. Our work experimentally realizes a reversible, wide range, low power consumption manner to controls and even enhances the spin Hall angle in sputtered Bi_2_Se_3_. Such controllable manipulation of spin Hall angle with exceptionally large enhancement utilizing electric field points to opportunities to enable energy-efficient SOT switching and tunable spintronic devices.

## Methods

### Film growth

Substrate/Bi_2_Se_3_(8 nm)/NiFe(8 nm)/TaO_*x*_(1 nm) multilayers were deposited onto thermally oxidized Si and (011)-oriented PMN-PT substrates by DC magnetron sputtering at room temperature with a base pressure less than 1 × 10^−7^ Torr. The background Ar working pressure was 3 mTorr for depositing all the layers. An in situ quartz crystal microbalance monitored the deposition rates.

### Device fabrication

The multilayer stacks were patterned into cross Hall bars and rectangular bars for planar Hall measurement and ST-FMR measurement respectively using standard maskless ultraviolet photolithography and lift-off procedure. The second step of photolithography was employed to fabricate the Cr(5 nm)/Au(100 nm) contacts.

### Characterization

The planar Hall measurements were performed with Keithley 6220 DC current source and Keithley 2812 nanovoltmeter in a homemade probe system. A Lake Shore Model 425 Gaussmeter monitored the magnetic field. The planar Hall resistance was recorded as the magnet was rotated. The in situ electric field across the PMN-PT was applied by Keithley 6517B electrometer. A cross-sectional TEM sample was prepared by the focused ion beam (FIB, Tescan LYRA 3). The crystal structure of the film was investigated using a probe aberration-corrected scanning transmission electron microscopy (Cs-STEM, Themis Z G2 300 kV, FEI). All of the measurements were performed at room temperature.

### DFT calculations

Our calculations are performed using the Vienna ab initio Simulation Package (VASP) based on the DFT^37,38^. The projector augmented wave method and a plane-wave basis set are performed. The electron exchange-correlation functional is described by the generalized gradient approximation of the Perdew–Burke–Ernzerhof functional and the HSE06 hybrid functional^42–44^. The plane-wave cutoff energy is chosen to be 500 eV. Moreover, at Γ-centered *k* mesh of 24 × 24 × 3 are adopted in the self-consistent calculations. To better describe the vdW interaction, the optB86b-vdW functional is adopted^45,46^. Then, DFT wave functions are projected to maximally localized Wannier functions using the WANNIER90 package^39^, and the Kubo formula is performed to calculate the SHC. Dene k points meshes of 100 × 100 × 100 and 150 × 150 × 150 are employed respectively for the cases without charge doping and with charge doping, to perform the Brillouin zone integration for the SHC calculations.

The Kubo formula for SHC is given by

$${\sigma }_{{\alpha }{\beta }}^{{\gamma }}=-\frac{{e}^{2}}{\hslash }\frac{1}{V{N}_{{k}}^{3}}\mathop{\sum} \limits_{k}{\varOmega }_{\alpha \beta }^{{\gamma }}(k)$$

${\sigma }_{\alpha \beta }^{\gamma }$ is the k-resolved term, which is written as^38,45^

$$\mathop{\sum} \limits_{k}{\varOmega }_{\alpha \beta }^{\gamma }(k)=\mathop{\sum} \limits_{n}{f}_{nk}{\varOmega }_{n,\alpha \beta }^{\gamma }(k)$$

where *f*_*nk*_ is the Fermi–Dirac distribution function and ${\varOmega }_{n,\alpha \beta }^{\gamma }(k)$ is the band-projected spin Berry curvature term written as

$${\varOmega }_{n,\alpha \beta }^{\gamma }(k)={\hslash }^{2}\mathop{\sum} \limits_{m\ne n}\frac{-2\,{{{\mbox{Im}}}}\,[\langle nk|\frac{1}{2}\{{\hat{\sigma }}_{\gamma },{\hat{v}}_{\alpha }\}|mk\rangle \langle mk|{\hat{v}}_{\beta }|nk\rangle ]}{{(\varepsilon {}_{nk}-\varepsilon {}_{mk})}^{2}}$$

where *α* denotes the spin current direction, *β* represents the direction of the applied electrical field, and *γ* shows the spin direction of the spin current. In addition, *V* is the cell volume and ${N}_{k}^{3}$ is the number of *k* points in the BZ.

### Reporting summary

Further information on research design is available in the Nature Research Reporting Summary linked to this article.

## Supplementary information

Supporting Information

Reporting Summary

## Data Availability

The data that support the findings of this study are available from the corresponding author on reasonable request.

## Code availability

The codes are available from the corresponding author upon reasonable request.
