# Supplementary material for: Giant tunable spin Hall angle in sputtered Bi2Se3 controlled by an electric field
Source: Nat Commun. 2022 Mar 28;13:1650. doi: 10.1038/s41467-022-29281-w (PMC8960771; doi:10.1038/s41467-022-29281-w)
Supplement: Supplementary file 1 — Supporting Information [file 41467_2022_29281_MOESM1_ESM.pdf]

SUPPLEMENTARY INFORMATION

Giant Tunable Spin Hall Angle in Sputtered Bi_2Se_3 Controlled by an Electric Field

Qi Lu [a#], Ping Li [b#], Zhixin Guo [b*], Guohua Dong [a*], Bin Peng [a], Xi Zha [a], Tai Min [b], Ziyao Zhou [a] and Ming Liu [a*]

[a] Electronic Materials Research Laboratory, Key Laboratory of the Ministry of Education & International Center for Dielectric Research, School of Electronic and Information Engineering, State Key Laboratory for Manufacturing System Engineering, Xi'an Jiaotong University, Xi'an 710049, China

[b] Center for Spintronics and Quantum System, State Key Laboratory for Mechanical Behavior of Materials, School of Materials Science and Engineering, Xi'an Jiaotong University, Xi'an 710049, China

*To whom correspondence should be addressed: zxguo08@xjtu.edu.cn, guohuadong@xjtu.edu.cn, mingliu@xjtu.edu.cn

[#] These authors contributed equally.

1. **Spin-torque characterization using ferromagnetic resonance**
2. **Magnetization of NiFe(8 nm)/Bi_2Se_3(8 nm) thin films**
3. **Resistivity measurement and current distribution**
4. **Energy dispersive X-ray spectroscopy (EDX) elemental mapping**
5. **XRD spectra for 200 nm Bi_2Se_3 film on Si substrate**
6. **Full XPS spectrum for the Bi_2Se_3 film**
7. **Characterization of chemical stoichiometry for the Bi_2Se_3 film**
8. **Characterization of the surface roughness on PMN-PT and Si substrate**
9. **Determination of the Oersted field**
10. **Charge density difference of heterostructure by HSE06 functional**
11. **The influence of Se vacancy defect and metal layer for charge transfer**
12. **The averaged charge density difference of Fe/Bi_2Se_3 heterostructure**

Supplementary Note 1. Spin-torque characterization using ferromagnetic resonance

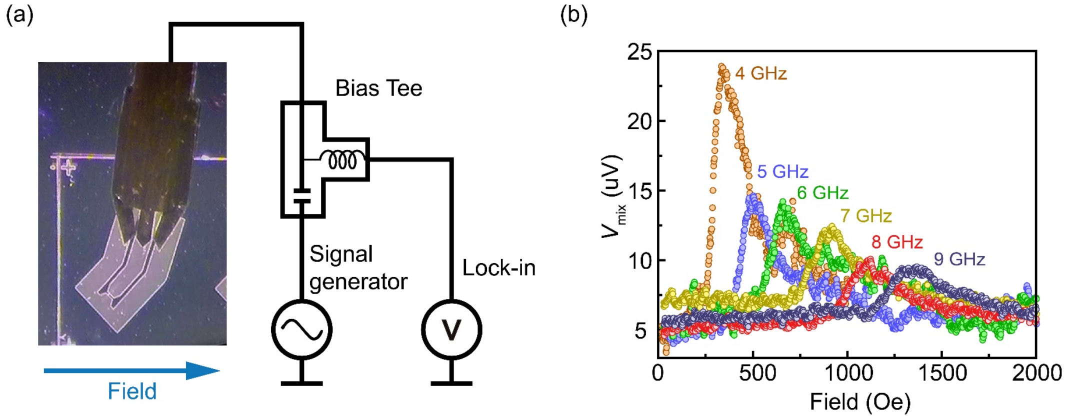

Supplementary Figure 1. (a) Optical micrograph of the NiFe(8 nm)/Bi_2Se_3(8 nm) device with GSG connection and the electrical circuit for the ST-FMR measurement. (b) ST-FMR spectra measured at different frequencies from 4 GHz to 9 GHz.

The ST-FMR measurement has been used as another technique to determine the spin Hall angle independently from the planar Hall measurement. The device layout and the measuring circuit are illustrated in Supplementary Fig. 1(a). The NiFe(8 nm)/Bi_2Se_3(8 nm) thin film grown on a thermally oxidized Si substrate is patterned into rectangular bars with dimension 5 μm (wide)*50 μm (long). Ground-Source-Ground (GSG) electrodes are used to inject the microwave signal and detect the DC mixing signal. An in-plane magnetic field is applied with an angle of 45° relative to the current sweeping from 0 Oe to 2000 Oe. The microwave current with the power of 10 dBm is modulated with a 437 Hz low frequency signal to detect the mixing voltage V_{mix} measured by a lock-in amplifier. Supplementary Fig. 1(b) shows the ST-FMR spectra

at a frequency range of 4 GHz to 9 GHz. The mixing voltage of the ST-FMR can be expressed as:

$$V_{\text{mix}} = S\frac{\Delta H^2}{(H-H_{\text{r}})^2+\Delta H^2} + A\frac{(H-H_{\text{r}})\Delta H}{(H-H_{\text{r}})^2+\Delta H^2} + \text{offset} \quad (1)$$

here the H_{r} represents the ferromagnetic resonance field and ΔH represents the ferromagnetic resonance linewidth. S and A are the magnitudes of the symmetric and antisymmetric components of the Lorentzian function. The parameters above will be obtained via the best fitting of the experimental results using Supplementary Equation (1).

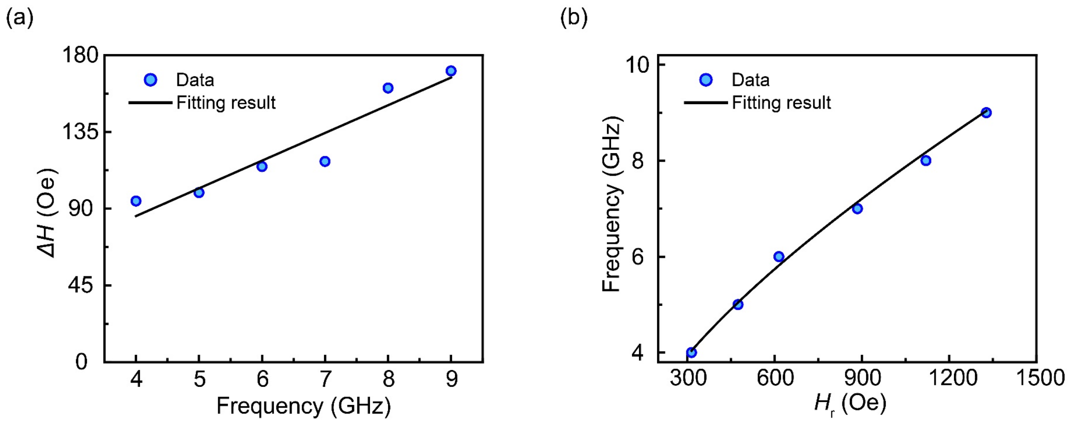

Supplementary Figure 2. (a) The FMR linewidth determined from ST-FMR signals at different frequencies. The Gilbert damping constant α is derived from the linear fitting result. (b) The FMR resonance field as a function of the microwave frequency. The solid curve represents a fit to the Kittel formula.

The derived ferromagnetic resonance linewidth ΔH versus frequency f is plotted in Supplementary Fig. 2(a). The Gilbert damping constant α can be calculated by the linear fitting using equation:

$$\Delta H = \Delta H_0 + \frac{4\pi\alpha f}{|\gamma|} \quad (2)$$

where γ represents the gyromagnetic and ΔH_0 is the inhomogeneous linewidth. The Gilbert damping constant α is determined to be 0.024±0.003. Moreover, the resonance field as a function of frequency are fit to the Kittel equation:

$$\left(\frac{2\pi f}{\gamma}\right)^2 = (H_r + H_k + 4\pi M_{eff})(H_r + H_k) \quad (3)$$

The value of $4\pi M_{eff}$ obtained from the Kittel equation is 5834±266 Oe, which is in general agreement with the result of magnetic hysteresis loop.

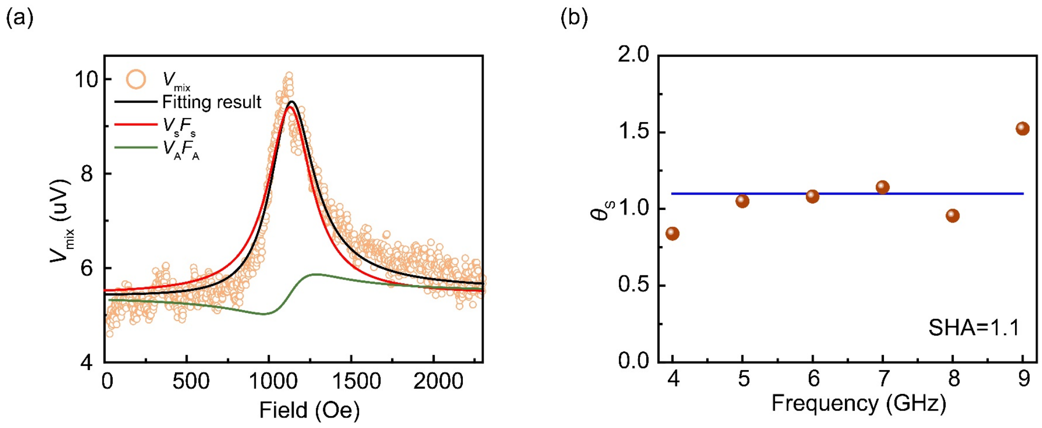

Supplementary Figure 3. (a) Typical ST-FMR spectrum recorded at 8 GHz. The hollow points represent experimental data, whereas the solid lines are Lorentzian line-shape fits separately with symmetric (red line) and antisymmetric (green line) components. (b) Spin Hall angle of 8 nm Bi_2Se_3 derived from the ratio of the symmetric and antisymmeteric components as a function of frequency.

Supplementary Fig. 3(a) shows a representative result of ST-FMR spectrum measured at 8 GHz. The V_{mix} signal has been well fitted into the overlap of symmetric Lorentzian line shape and antisymmetric Lorentzian line shape. It is notable that the

symmetric Lorentzian component dominates the total ST-FMR spectrum, corresponding to a large damping-like effective field H_{DL}. The FMR spin-torque generation efficiency can be quantitatively determined by the ratio of symmetric component and antisymmetric component S/A obtained from the resonance line shape. (line-shape coefficients S and A)

$$\xi_{FMR}=\frac{S}{A}\frac{e\mu_0 M_{\mathrm{S}} d_{\mathrm{F}} d_{\mathrm{N}}}{\hbar}\sqrt{1+\frac{4\pi M_{\mathrm{eff}}}{H_{\mathrm{r}}}} \quad (4)$$

where $\hbar$ represents the reduced Planck's constant, d_{F} and d_{N} is the thickness of the ferromagnetic layer and nonmagnetic layer respectively. The SOT efficiency ξ calculated under different excitation frequencies are plotted in Supplementary Fig. 3(b). The sputtered Bi_2Se_3 exhibits a large SOT efficiency around 1.1 measured by ST-FMR, which is comparable with the result of planar Hall measurements.

Supplementary Note 2. Magnetization of NiFe(8 nm)/Bi_2Se_3(8 nm) thin films

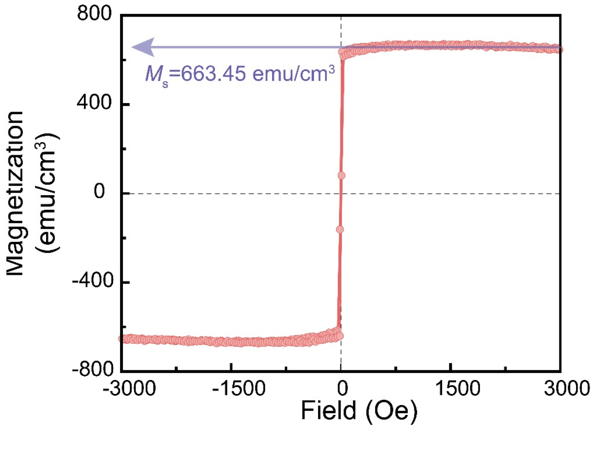

Supplementary Figure 4. Magnetization hysteresis loop recorded on the NiFe(8 nm)/Bi_2Se_3(8 nm) full film.

The NiFe(8 nm)/Bi_2Se_3(8 nm) full film grown on a 5 mm×5 mm thermally oxidized Si substrate in the same batch with the Hall device is used to determine the saturation magnetization. The magnetic hysteresis loop measured by the vibrating sample magnetometer is shown in Supplementary Fig. 4. The magnetization curve shows that the coercive field is less than 10 Oe. Hence the 400 Oe external magnetic field is enough to ensure the magnetization oriented along the external field. Using the measured volume of the full film, the saturation magnetization M_s is calculated to be 663.45 emu/cm^3.

Supplementary Note 3. Resistivity measurement and current distribution

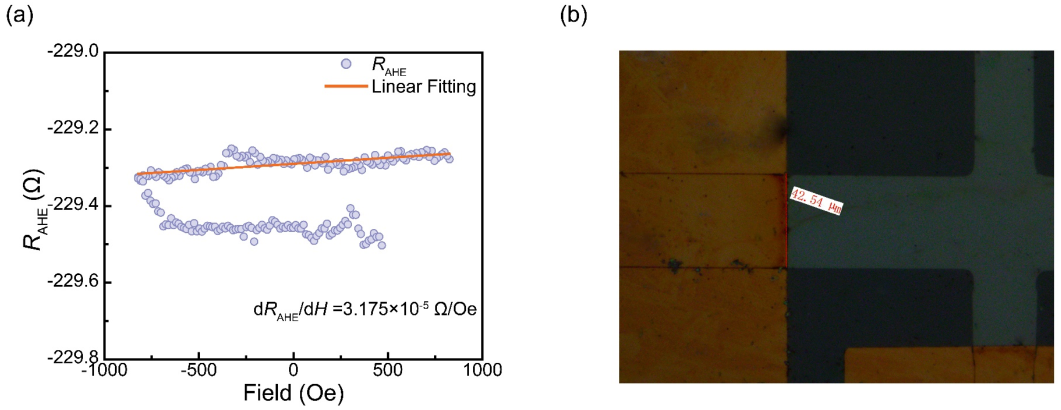

Supplementary Figure 5. (a) Anomalous Hall resistance R_{AHE} vs H_{perp} for sample PMN-PT/Bi_2Se_3(8 nm)/NiFe(8 nm)/TaO_x(1 nm). (b) Optical image of the Hall cross region in the measured device.

The resistivity of each layer is essential to determine the charge current density in the Bi_2Se_3 spin Hall layer. The resistivity of 8 nm NiFe thin film and 8 nm Bi_2Se_3 thin film are accurately measured in a typical four-probe mode set up by Keithley 6220 DC

current source and Keithley 2812 nanovoltmeter with 1 mA testing current. After the film deposition, the resistivity is immediately recorded to minimize the metal oxidation. The resistivity of 8 nm NiFe thin film is 52.71± 0.15 μΩ cm and 8 nm Bi_2Se_3 thin film is 1118.18 ± 3.87 μΩ cm. To determine the value of $\frac{dR_{\mathrm{AHE}}}{dH_{\mathrm{perp}}}$, the anomalous Hall resistance was measured against the out-of-plane field H_{perp} under 1 mA small testing current. Here $\frac{dR_{\mathrm{AHE}}}{dH_{\mathrm{perp}}}$ is obtained to be 3.175×10^{-5} Ω/Oe by taking the slope of R_{AHE} versus H_{perp} as shown in Supplementary Fig. 5(a). Moverover, the width of the Hall bar is calibrated to be 42.54 μm by an optical microscope as shown in Supplementary Fig. 5(b). The current distribution in each layer can be calculated by the current shunting equation.

Supplementary Note 4. Energy dispersive X-ray spectroscopy (EDX) elemental mapping

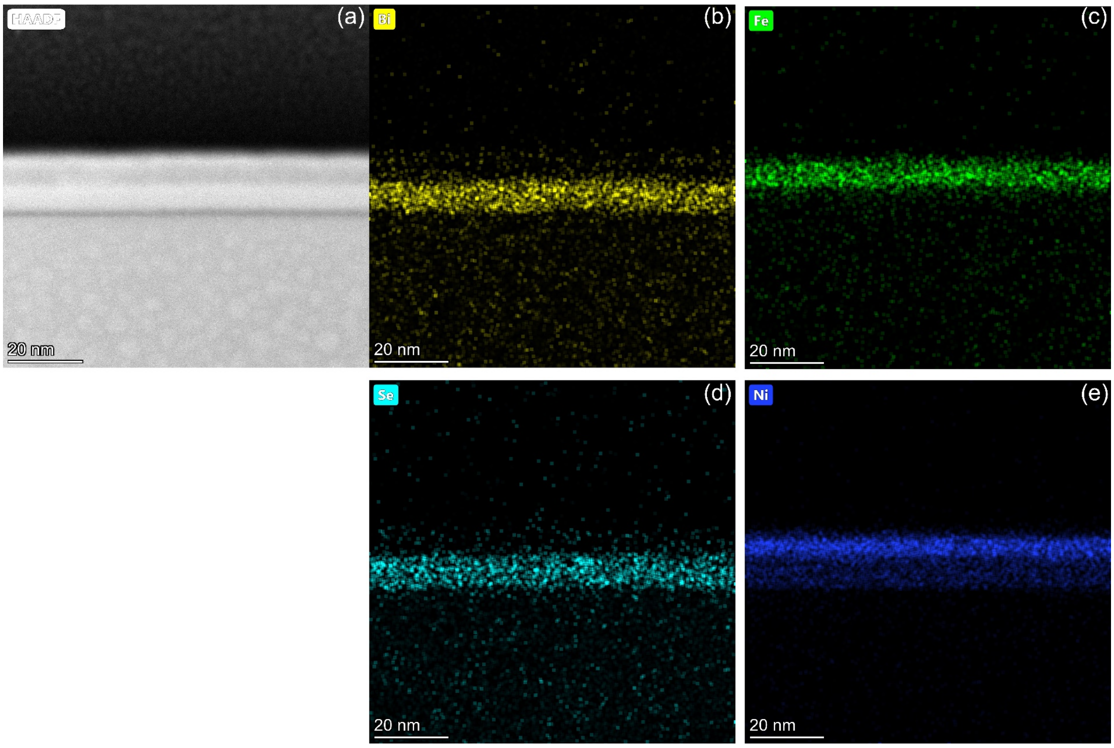

Supplementary Figure 6. (a) Cross-sectional HAADF-STEM image for sample PMN-PT/Bi_2Se_3(8 nm)/NiFe(8 nm)/TaO_x(1 nm). (b), (c), (d) and (e) Corresponding elemental EDX mapping for Bi, Fe, Se and Ni respectively.

To get more concrete insights into the Bi_2Se_3/NiFe interface, HAADF-STEM image and corresponding elemental maps for Bi, Fe, Se and Ni were shown in Supplementary Fig. 6. It can be seen that almost all of the layers are well separated. Elemental mapping of the stack supports the uniform growth of Bi_2Se_3 layer and NiFe layer in the stack.

Supplementary Note 5. XRD spectra for 200 nm Bi_2Se_3 film on Si substrate

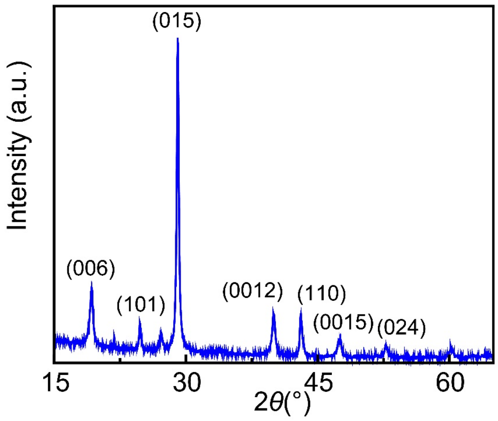

Supplementary Figure 7. XRD spectrum for 200 nm Bi_2Se_3 film on Si substrate.

To further verify the crystallinity of our sputtered Bi_2Se_3 film, we deposit 200 nm Bi_2Se_3 film on a thermally oxidized Si substrate for the XRD measurement as shown in Supplementary Fig. 7. The film is polycrystalline and the diffraction peaks can be exactly indexed by the standard diffraction planes of R-3m hexagonal Bi_2Se_3, JCPDS No. 33-0214.

Supplementary Note 6. Full XPS spectra for the Bi_2Se_3 film

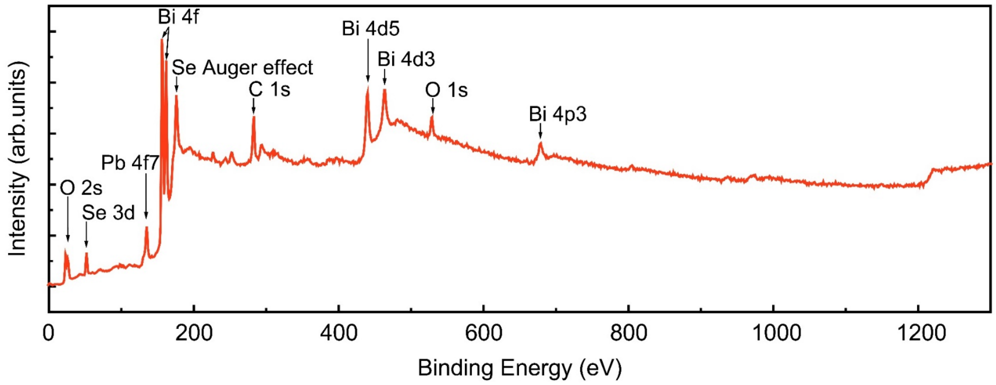

Supplementary Figure 8. Full XPS spectra for the 20 nm Bi_2Se_3 film on the PMN-PT substrate.

The full XPS spectra of 20 nm Bi_2Se_3 film on PMN-PT substrate is shown in Supplementary Fig. 8. The energy level of each peak is labeled one by one in the figure. We find that the element in the spectrum are: Bi, Se, Pb(from PMN-PT substrate) with little impurities of O and C which may come from the air atmosphere.

Supplementary Note 7. Characterization of chemical stoichiometry for the Bi_2Se_3 film

We calculate the stoichiometry from the XPS spectra illustrated in Supplementary Fig. 8. The composition ratio of element can be obtained by the following expression:

$$\left(\frac{n_a}{n_b}\right)=\frac{I_a / S_a}{I_b / S_b} \tag{5}$$

where I is the peak area and S is the relative sensitivity factor. Here we select Bi $4p_{3/2}$ peak and Se 3d peak to calculate the composition ratio due to the relative horizontal baseline.

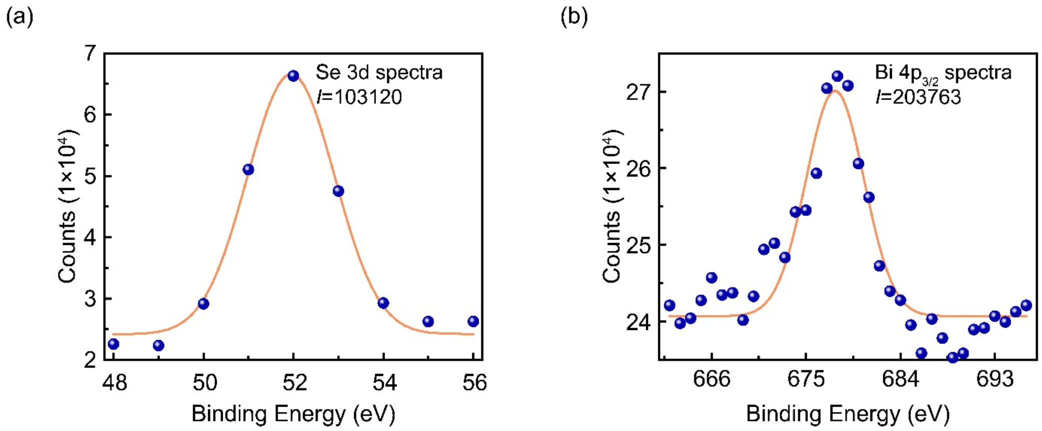

Supplementary Figure 9. XPS spectra of Se 3d and Bi $4p_{3/2}$ core level for the calculation of element ratio.

As shown in Supplementary Fig. 9, the peak areas are calculated by the fitting of standard Gaussian lineshape. It is found that the area of Bi $4p_{3/2}$ peak and Se 3d peak

are 203763 and 103120, respectively. The corresponding relative sensitivity factor of Bi $4p_{3/2}$ peak is 6.48 and Se 3d peak is 2.29, respectively. Thus, the atomic composition ratio of Se and Bi is estimated to be 1.43, which is very close to the stoichiometry of target Bi_2Se_3. Note that XPS can provide only the relative rather than the high accuracy ratios of elements in a sample. Hence, our result is within the error bar of measurement, which shows that our Bi_2Se_3 films are normally stoichiometric.

Supplementary Note 8. Characterization of the surface roughness on PMN-PT and Si substrate

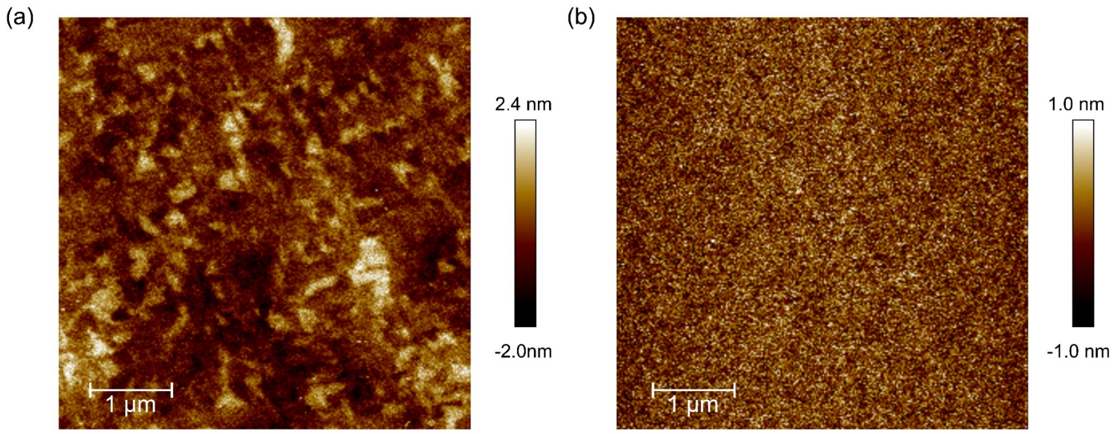

Supplementary Figure 10. AFM image of (a) Bi_2Se_3/NiFe/TaO_x film on PMN-PT substrate and (b) Si substrate.

With regarding to the surface roughness, we conducted the AFM measurements on the Bi_2Se_3(8 nm)/NiFe(8 nm)/TaO_x(1 nm) films, which are deposited on PMN-PT substrate and thermally oxidized Si substrate, respectively. As shown in Supplementary Fig. 10, the surface roughness Ra of the sample on PMN-PT substrate is 0.49 nm and the sample on Si substrate is 0.24 nm. The pattern in the image of PMN-PT substrate is induced by the ferroelastic domain in the PMN-PT, which is a common feature when measuring the AFM image on a ferroelectric substrate [Adv. Mater. 25, 4886 (2013), ACS Appl. Electron. Mater. 1, 1012 (2019), J. Appl. Phys. 124, 173103 (2018)]. The PMN-PT substrate shows a relatively high roughness than thermally oxidized Si

substrate because of the different polishing processes. Nevertheless, all the samples show a smooth surface and high film quality that is sufficient to further SOT study.

Supplementary Note 9. Determination of the Oersted field

Since the width of Hall bar is much larger than the thickness of Bi_2Se_3 layer, the sample can be approximated as an infinitely wide conducting plate. In this case the current induced Oersted field can be derived from the Biot-Savart Law as[Science 336, 555 (2012)]:

$$H_{\mathrm{Oe}} = \frac{\mu_0 J(d_1 + d_2)}{2} \quad (6)$$

where μ_0 is magnetic permeability in a vacuum, J is the current density through the Bi_2Se_3 layer, d_1 and d_2 represent the thickness of Bi_2Se_3 layer and NiFe layer respectively. When the 1 mA current inject into the whole device, the current density through Bi_2Se_3 is about 6.62×10^7 A/m^2 after considering the current shunting effect. Therefore we get that the Oersted field generated by 1mA current is 6.66×10^{-3} Oe.

Supplementary Note 10. Charge density difference of heterostructure by HSE06 functional

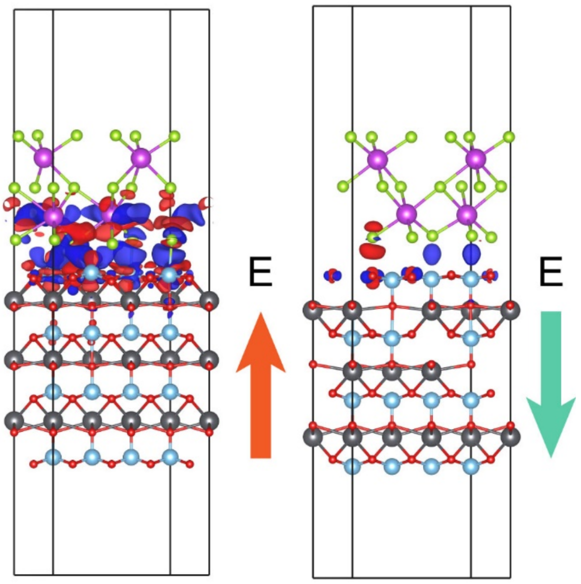

Supplementary Figure 11. Charge density difference of heterostructure of Bi_2Se_3 on TiO-terminal $PbTiO_3$(001) surface calculated using HSE06 functional. The left (right) panel corresponds to the results when a negative (positive) electric field is applied in the experiment. The direction of the electric field is indicated by the arrows. The red and blue areas indicate loss and gain electrons, respectively.

Supplementary Note 11. The influence of Se vacancy defect and metal layer for charge transfer

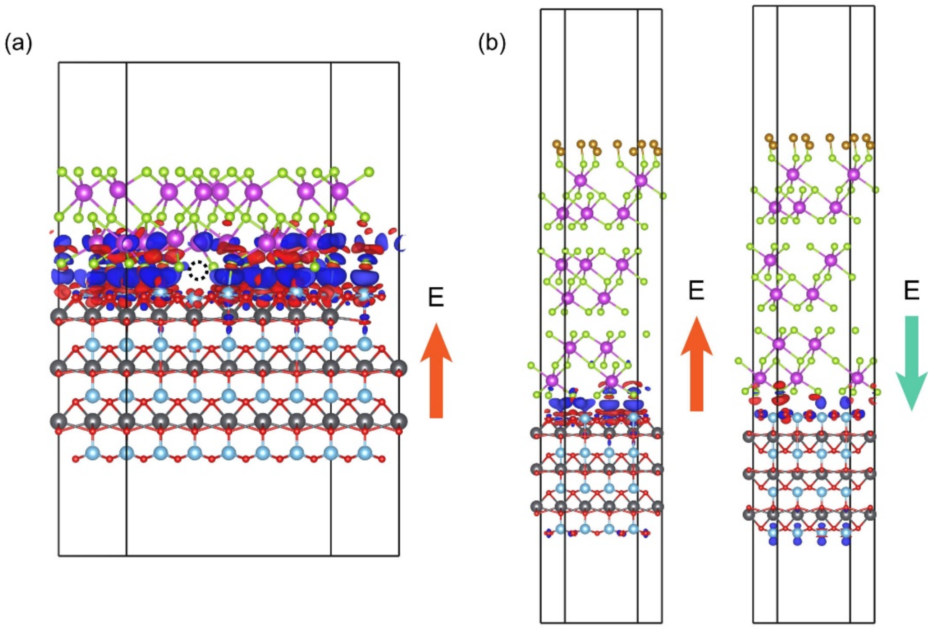

Supplementary Figure 12. Charge density difference of $Bi_2Se_3/PbTiO_3$ heterostructure with Se vacancy defect at the interface. (a) Charge density difference of $Bi_2Se_3/PbTiO_3$ heterostructure under negative electric field (the direction is from $PbTiO_3$ toward Bi_2Se_3). The dotted circle represents Se vacancy. (b) Charge density difference of $Fe/Bi_2Se_3/PbTiO_3$ heterostructure. The left and right panels correspond to the cases with the negative and positive external electric fields, respectively. The red and blue areas indicate loss and gain electrons, respectively. The Arrows in the figures additionally show the direction of external electric fields.

Supplementary Note 12. The averaged charge density difference of Fe/Bi_2Se_3 heterostructure

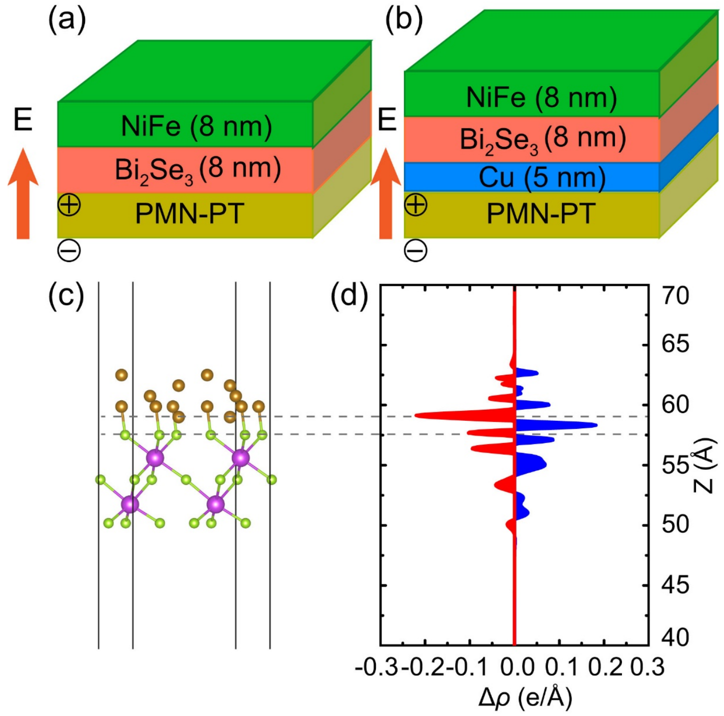

Supplementary Figure 13. (a, b) Schematics of the experimental setup for the voltage control spin Hall angle. (c) Side view of Fe/Bi_2Se_3 heterostructure. (d) Planar averaged charge density difference $\Delta\rho$ along z direction in the Fe/Bi_2Se_3 heterostructure. The positive (blue) and negative (red) $\Delta\rho$ values in (d) indicate the charge accumulation and depletion, respectively.
